# Poster Session II - A202 OPTIMIZING CARE: DISCONTINUING EMPIRIC PROTON PUMP INHIBITORS IN PATIENTS WITHOUT EVIDENCE OF GASTROESOPHAGEAL REFLUX DISEASE (GERD)

**DOI:** 10.1093/jcag/gwaf042.202

**Published:** 2026-02-13

**Authors:** K J Comishen, D M Rodrigues

**Affiliations:** Queen’s University, Kingston, ON, Canada; Queen’s University, Kingston, ON, Canada

## Abstract

**Background:**

Proton pump inhibitors (PPI) are one of the most prescribed class of drugs in Canada. The high prevalence of PPI use is associated with the empiric treatment of gastroesophageal reflux disease (GERD) symptoms. However, not all patients with typical GERD symptoms have pathologic reflux of acid. The presence of esophageal mucosal injury and/or inappropriately elevated acid exposure time helps differentiate GERD from functional heartburn, where symptoms occur without pathological reflux. Continuing PPI therapy in patients without pathologic GERD provides minimal benefit and may expose these individuals to unnecessary risks, including nutrient deficiencies, increased susceptibility to gastrointestinal infections, and kidney injury. Deprescribing empiric PPI therapy, therefore, is an important step in managing patients found to not have GERD.

**Aims:**

The goal of this study was to assess what percentage of patients referred to Kingston Health Sciences Center (KHSC) with suspected GERD on empiric PPI therapy have their PPI discontinued following normal endoscopic and acid reflux monitoring studies.

**Methods:**

A retrospective cross-sectional study was conducted. Participants were adult patients with suspected GERD on PPI therapy who were referred to KHSC between January 2023 and December 2024 for 24h ambulatory pH studies following normal endoscopy studies conducted off PPI therapy. Patients that successfully completed the 24h ambulatory pH studies off PPI therapy were diagnosed with GERD or not. The primary outcome was PPI use following disclosure of the pH study results to patients. Continued PPI use was determined by a combination of chart review and patient interviews.

**Results:**

A total of 56 patients with suspected GERD on empiric PPI therapy that were referred to KHSC from January 2023 and December 2024 were identified for having completed both endoscopy and acid reflux monitoring while off PPI therapy. Of the 56 patients, 49 patients had their PPI use determined either by documentation on their medical charts and/or by telephone interview. A total of 35 patients had normal endoscopy and normal esophageal acid monitoring; of these, 21 (60%) remained on PPI therapy. This contrasted with 13 of 14 patients (93%) who remained on PPI therapy after being diagnosed with GERD due to abnormal 24h pH studies.

**Conclusions:**

Patients often remain on PPI therapy for reflux even when diagnostic investigations provide no supportive evidence of GERD. Given that PPI therapy is not benign and patient compliance appears high, it is important that physicians actively deprescribe PPI therapy in patients without evidence of GERD.

**Funding Agencies:**

None

